# Supplementary material for: Directional flows using capillary assembly of photo-deformable colloidal particles at water-air interfaces
Source: Nat Commun. 2025 Dec 24;17:1004. doi: 10.1038/s41467-025-67739-9 (PMC12847863; doi:10.1038/s41467-025-67739-9)
Supplement: Supplementary file 1 — Supplementary Information [file 41467_2025_67739_MOESM1_ESM.pdf]

Supplementary Information

Directional flows using capillary assembly of photo-deformable colloidal particles at water-air interfaces

David Urban[1,2,5✉], Marcel Rey[3,4], Antonio Ciarlo[4], Marie Friederike Schulte[3], Emiliano Descrovi[2✉] and Giovanni Volpe[4]

1. Department of Electronic Systems, Norwegian University of Science and Technology, O.S. Bragstads plass 2b, 7491, Trondheim, Norway

2. Dipartimento di Scienza Applicata e Tecnologia, Politecnico di Torino, Corso Duca degli Abruzzi 24, 10129, Torino, Italy

3. Institute of Physical Chemistry, University of Münster, Corrensstr. 28/30, 48149 Münster, Germany

4. Department of Physics, University of Gothenburg, SE-41296, Gothenburg, Sweden

5. Present Address: SINTEF Digital, P.O. Box 124 Blindern, 0314, Oslo, Norway

✉Corresponding Authors

David Urban: david.urban@sintef.no
Emiliano Descrovi: emiliano.descrovi@polito.it

SUPPLEMENTARY FIGURES

Supplementary Figure 1 – SEM particle size distributions

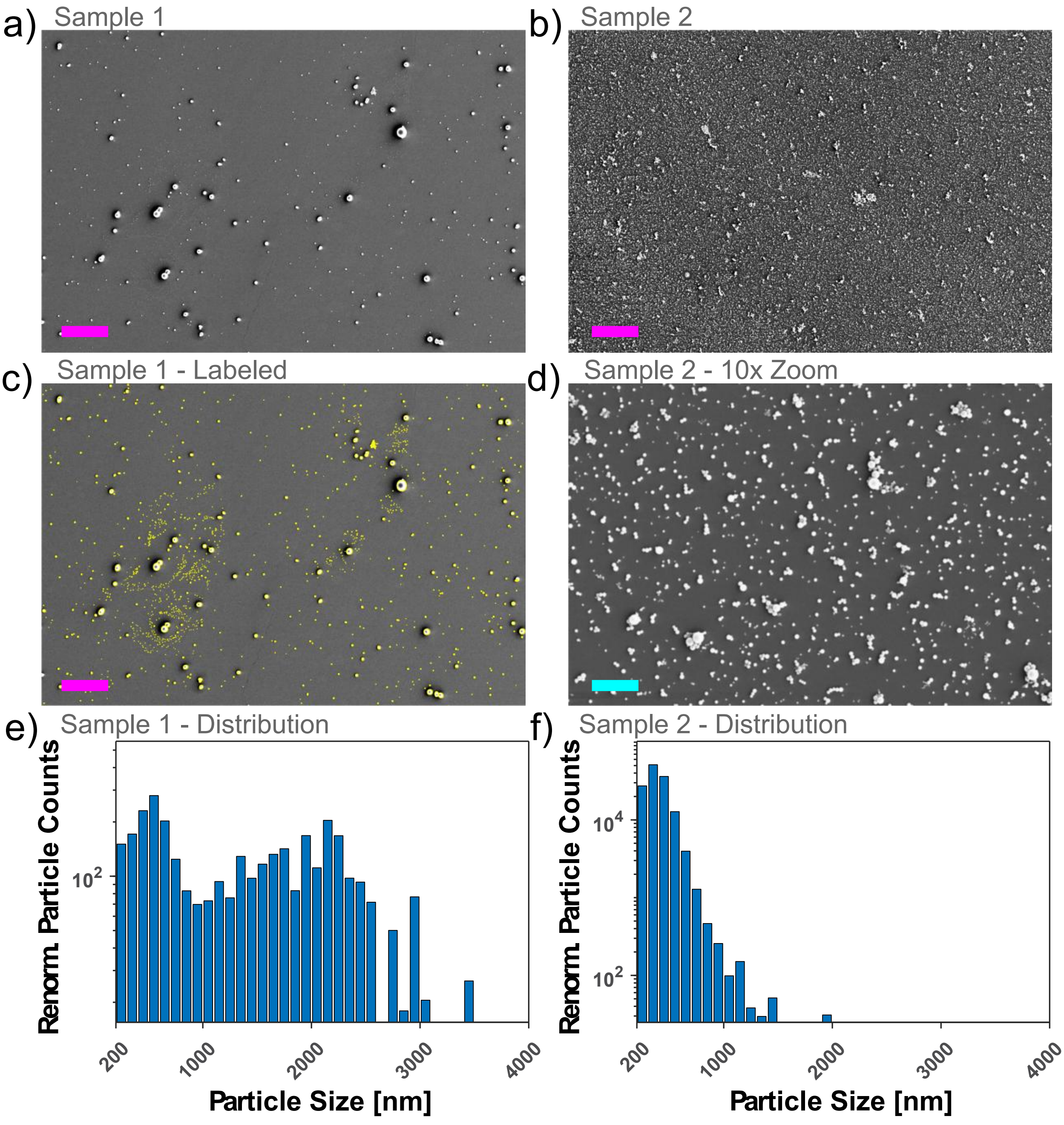

Supplementary Figure 1: SEM images of dilute, dried particles on a silicon substrate and particle counts. a/b Raw SEM images of Sample 1/Sample 2 respectively, with similar absolute content of azopolymer before drying. Note that sample 1 was employed in result sections 1-3, whilst Sample 2 was employed in result section 4. c) Example image of detected particle outlines (yellow) superimposed on raw image for Sample 1. Scale bars (magenta): 20 µm d) 10x larger zoom-in on Sample 1 particles, highlighting the presence of small particles. Scale bar (cyan): 2 µm. e/f) Particle counts based on 5 large area images, as in (a), for Sample1 and 3 large area images, as in (b), for Sample 2. Particle absolute counts were renormalized to the occupied cross-section in 2D at the interface.

Supplementary Figure 2 – Confocal z-stack of interface-adsorbed particles

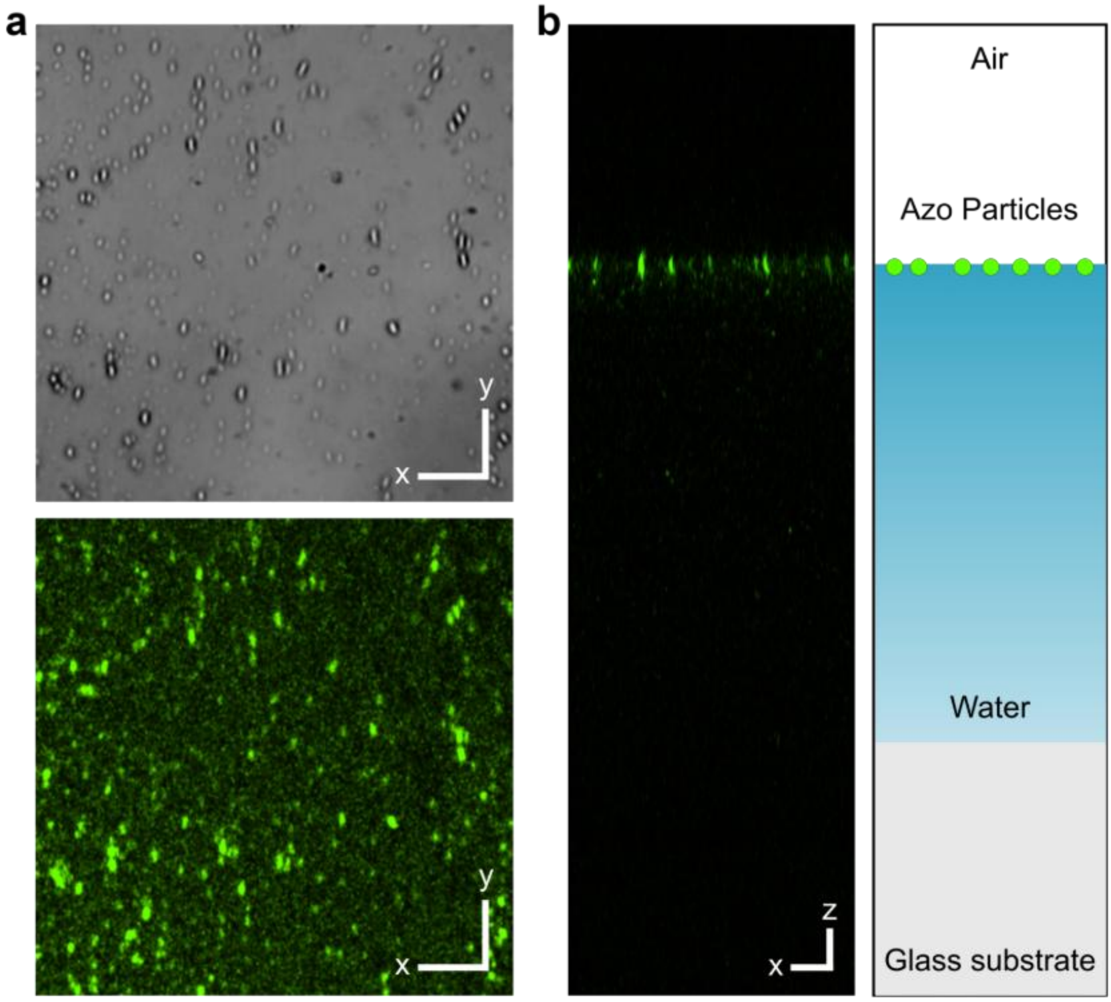

Supplementary Figure 2. Confocal stack of interface-adsorbed particles. a) Single 2D slice from a confocal scan, imaging the particles based on the azopolymer's intrinsic fluorescence, at the *z*-position of the water-air interface. Top: transmitted light detector. Bottom: fluorescent (confocal) signal. b) Full confocal *z*-stack through the sample cell illustrating that the majority of particles are confined at the liquid interface. Right: schematic illustration showing the different samples sections corresponding to the z-stack depth, with particles illustrated in green. All scale bars (white): 10 µm. Note that the particles become directionally deformed during imaging as described in the result section "Shape-morphing particles at an air-water interface", here in the *y*-direction corresponding to the linear polarization orientation of the confocal laser that is used for excitation (wavelength 514 nm). The directional deformations lead to capillary bonds as described in the result section "Optically controllable capillary interactions and assembly". These effects are particularly visible on the transmitted light image, whilst the presence of smaller particles (see Supplementary Figure 1) is more easily appreciated on the fluorescent one.

Supplementary Figure 3 – Three-phase contact angle by gel trapping

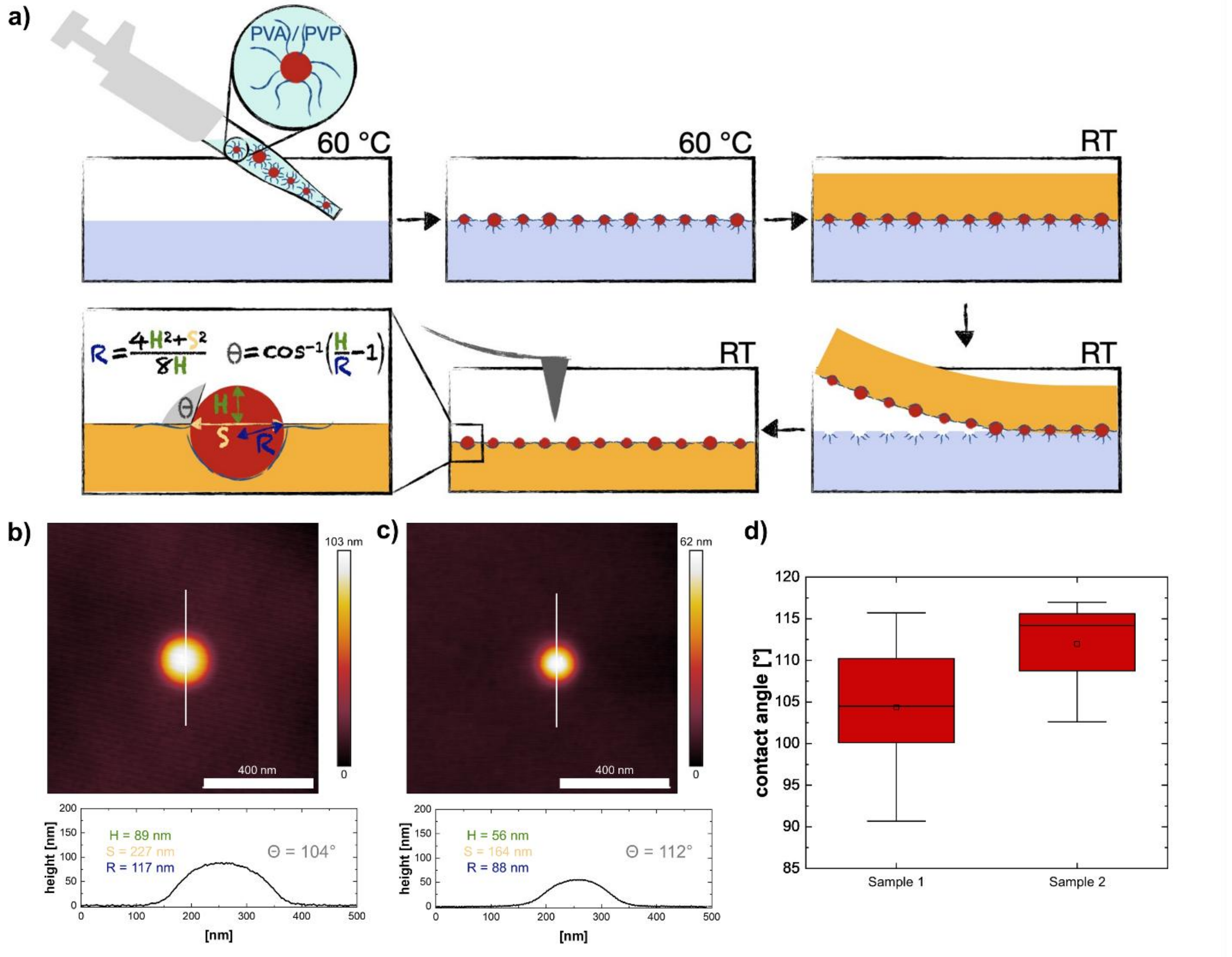

Supplementary Figure 3: a) Schematic illustration of the determination of the three-phase contact angle via the gel trapping technique developed by Paunov, with an atomic force microscope (AFM; for details see Methods). AFM height image with cross-section at the indicated white line for (b) Sample 1, and (c) Sample 2. d) Box plots of the determined contact angles for either sample type, based on n = 25 measurements for Sample 1 and n = 20 measurements for Sample 2. The red boxes delimit the interquartile range (between 25th and 75th percentile) of the measurement distributions in each case; the horizontal line marks their median and the square marks their mean. Whiskers mark the 5th and the 95th percentile of the distributions. Contact angle mean and sample standard deviation were 104° ± 6° for Sample 1 and 111° ± 4° for Sample 2 respectively. Note that Sample 1 was employed in result sections 1-3, whilst Sample 2 was employed in result section 4.

Supplementary Figure 4 – Single particle deformation quantification

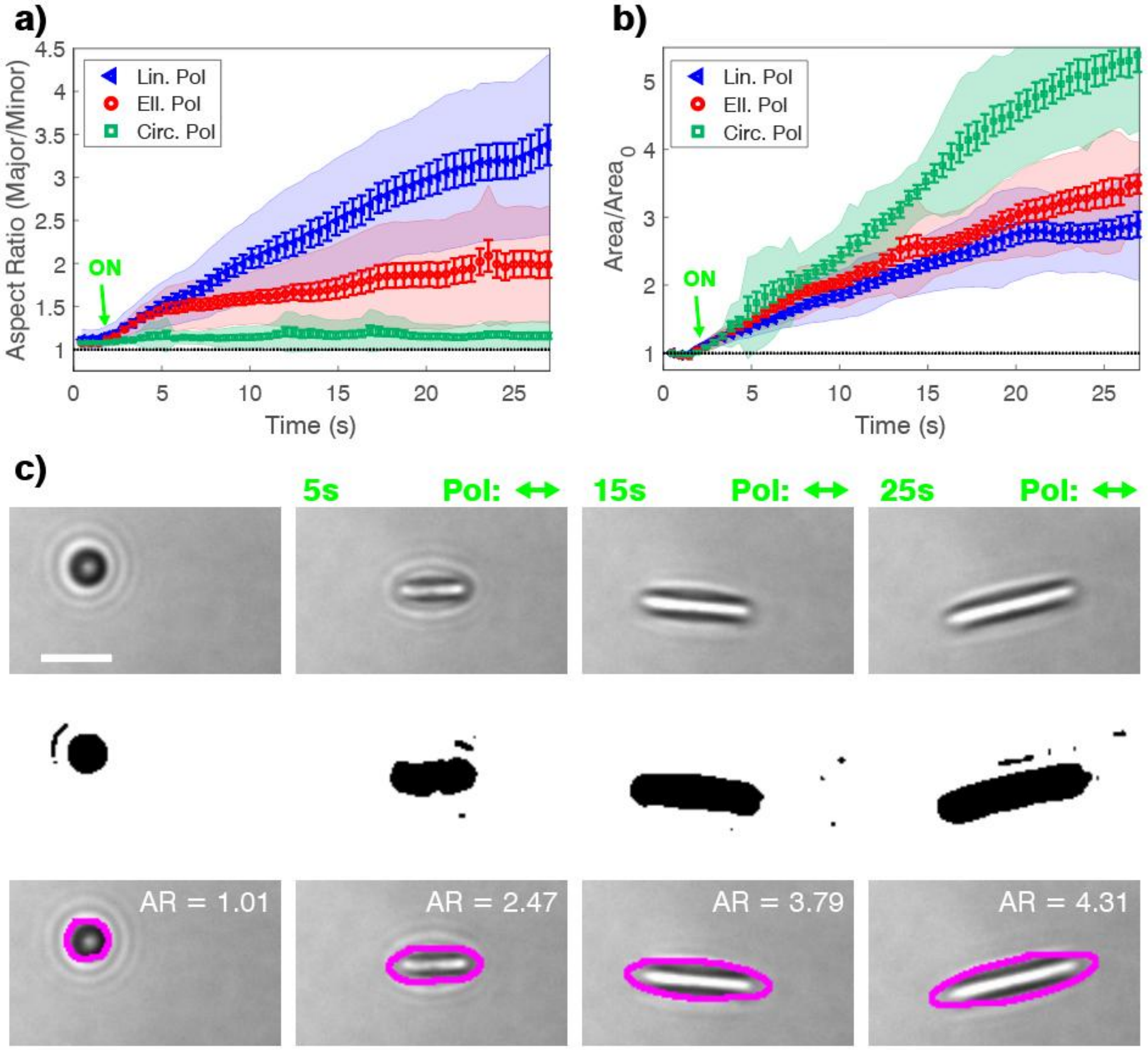

Supplementary Figure 4: a) Mean aspect ratio vs. time of particles at the interface under illumination with linearly, elliptically and circularly polarized light. As exemplified in Figure 1, the induced in plane aspect ratio is highest for linear polarization, while for circular polarization the value remains close to one. b) Similar graph, reporting the mean relative area expansion vs. time. Here, circular polarization is found to induce the largest net flattening, albeit considerable area expansion is also produced in the other cases. In both graphs, error bars report the standard error of the mean (s.e.m.) for 20 individual particles for each polarization, while the color-shaded regions represent the distribution width based on the corresponding standard deviation (s.d.). Note that while a clear statistical difference can be observed between the different sample populations, there is also a considerable distribution overlap, especially between linear and elliptical polarization. This can supposedly be explained by the polydisperse sizes of the particles, their random rotational diffusion under illumination as well as fitting noise. c) Example images at different time points of the fitting procedure for a movie showing a particle deformed with linear polarization. Top-to-bottom: movie frame, corresponding segmented frame, and movie frame with fitted ellipse overlay. The fitting procedure is detailed in the Method section. All stacks and corresponding segmented versions with values are included in the shared data folder. Intensity I = 219W/cm^2. Scale bar (white): 5μm.

Supplementary Figure 5 – Fast dynamics of tip-to-tip approach and rotation

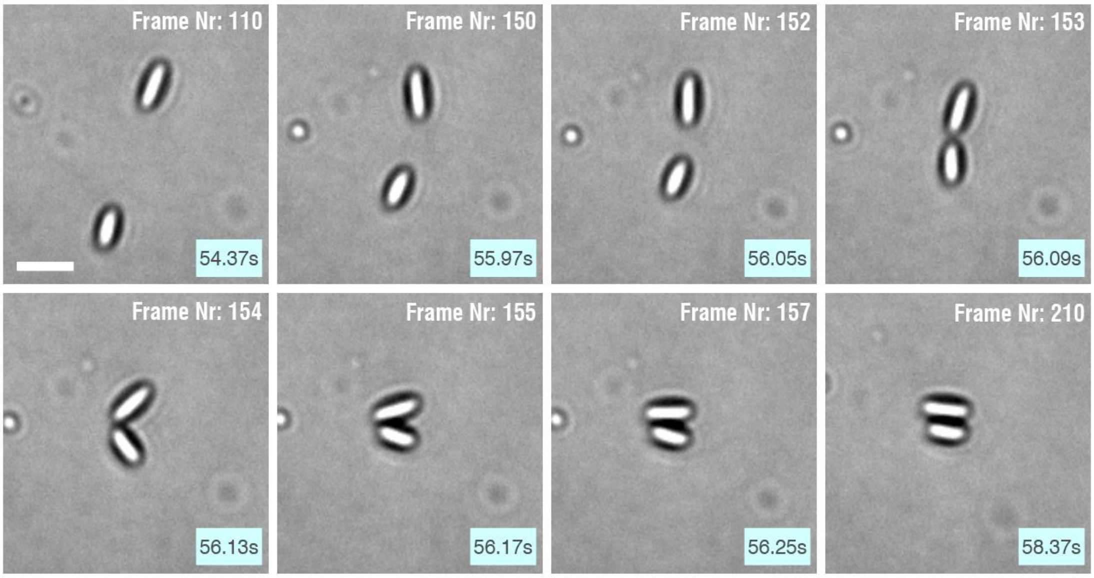

Supplementary Figure 5: Frames from Supplementary Movie 2 around the binding event (no illumination). The elliptical particles are seen to approach in a tip-to-tip configuration. Upon apparent contact, they rotate about the binding point within a fraction of a second. Scale bar (white): 5 µm. Light blue squares in the lower right indicate acquisition time.

Supplementary Figure 6 – Effective interparticle distances leading to assembly

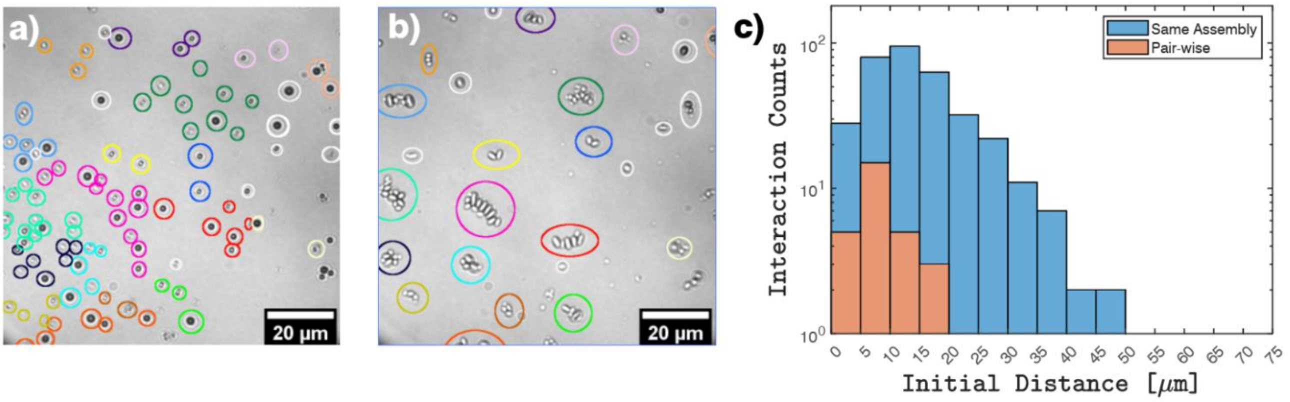

Supplementary Figure 6: Example quantifying all initial inter-particle distances leading to mutual assembly after a short (7s), elliptically polarized illumination with intensity I = 219 W/cm^2. a) Frame before illumination, with freely diffusing particles labeled with a different color for each of the 19 final, separate particle assemblies shown in (b). c) Plots of the initial distances between particles undergoing pair-wise assembly at an early stage, as well as initial distances between all particles ending up in the same final assembly. Here, pair-wise assemblies are observed up to separation distances of 20 µm and particles initially up to 50 µm apart are found to end up in the same assembly. While these distances are consistent with the orders of magnitude for effective interaction distances reported in literature, note that particle sizes and geometries will influence the interaction length. Hence, e.g., the illumination time and polarization used may influence the final measured result. The same amounts for the time interval up to the definition of "final assemblies" since assembly events (for example of smaller sub-assemblies) can be observed also after the illumination is terminated. Finally, the particle density can also play a role in defining the initial distances leading to common assembly. In dense particle settings, such as employed for example in Figure 3, the particles can supposedly more easily relay the capillary forces between each other, and the final assembly appears to span large parts of the illumination spot (~80 µm) as indicated by its strongly reduced diffusion visible when the laser is off. The full movie used for the empirical estimates shown here, the imageJ procedure of the segmentation of the frame in (a), and the segmented image as well as extracted positional values are included in the shared data folder.

Supplementary Figure 7 – Linear → Circular deformation sequence

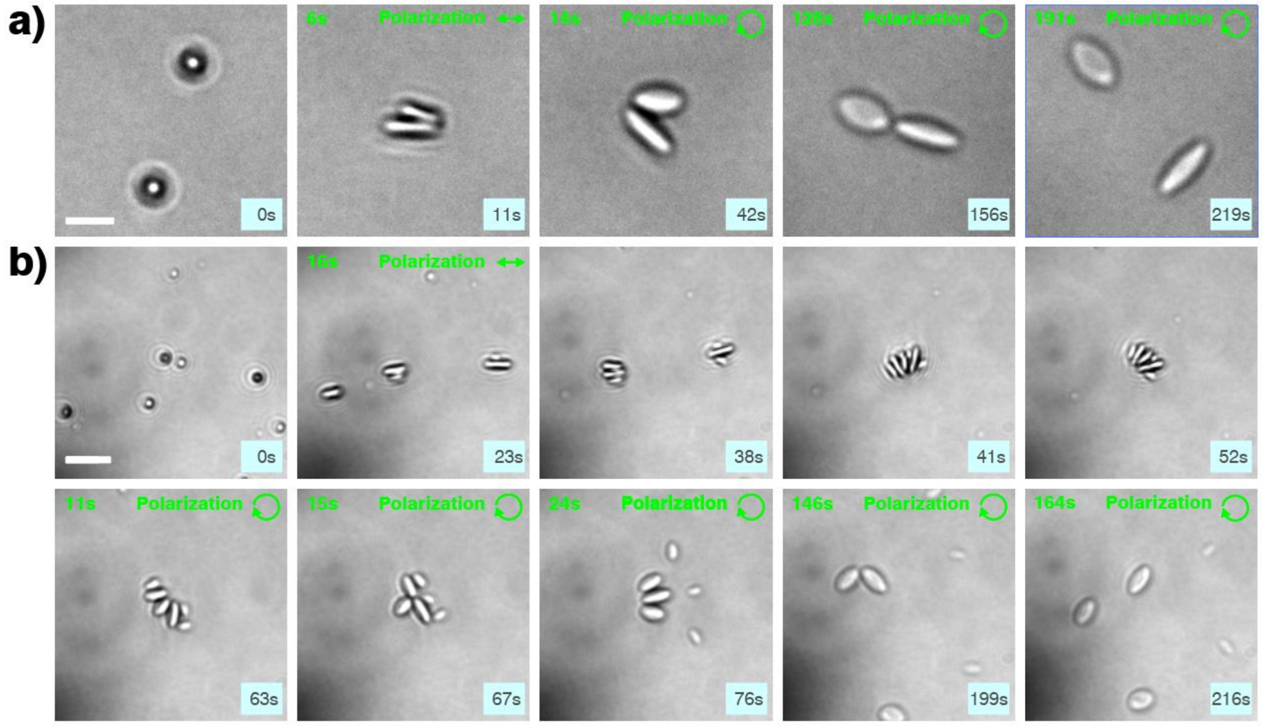

Supplementary Figure 7: a) Snapshots from Supplementary Movie 4, showing left-to-right: Deformation with linear polarization leading to rod-like shapes and consequential assembly, followed by prolonged illumination with circular polarization leading to increasing roundness and flattening and eventually to disassembly. Scale bar (white): 5 µm. b) Similar sequence involving multiple particles. Note that particles continue to assemble due to their rod-like shape, also after linear polarization has been switched off. They stay rigidly assembled until illumination with circular polarization remodels their shape. Full sequence is shown in Supplementary Movie 5. Scale bar (white): 10 µm. Light blue squares in the lower right indicate total acquisition time, green font indicates respective time from illumination onset.

Supplementary Figure 8 – No assembly with circular polarization

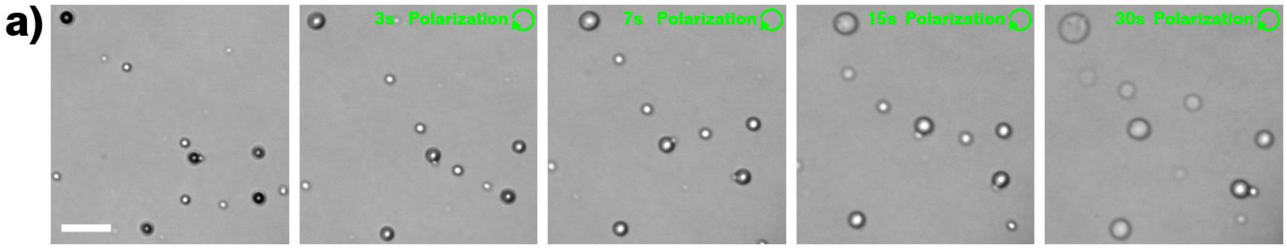

Supplementary Figure 8: a) Irradiation of pristine particles with circular polarization shown at different timepoints of illumination. The full movie is included as Supplementary Movie 6. Despite a similar density condition to Figure 2c/Supplementary Movie 3 (elliptical polarization) or Supplementary Figure 7b and Supplementary Movie 5 (linear polarization), no assembly is induced for circular polarization. The particles diffuse freely as their shapes are transformed into flattened disks. Intensity I = 219 W/cm^2, Scale bar (white): 10 µm.

Supplementary Figure 9 – Dynamic remodeling behavior during particle flattening.

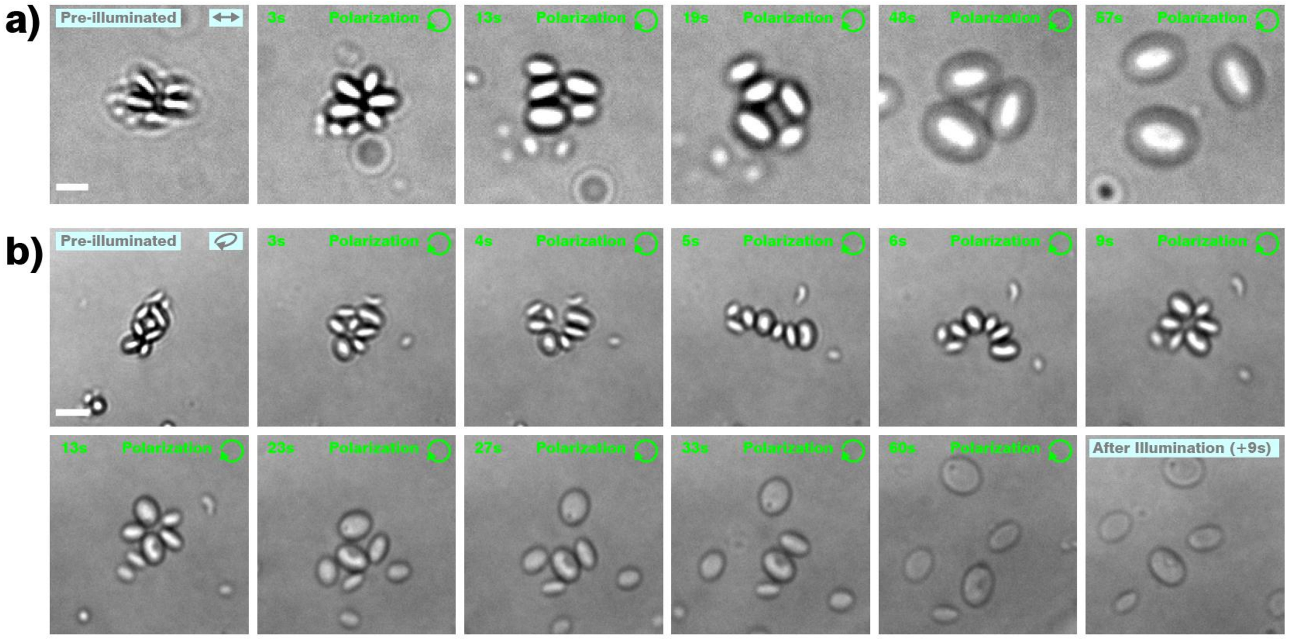

Supplementary Figure 9: a) Prolonged illumination of an optically pre-assembled particle structure with circularly polarized light. During the flattening of the particles the structure is continuously re-configurating itself, passing through symmetric flower-like, rectangular and finally triangular shapes before final disassembly. Scale bar (white): 3 µm. b) Example of another optically pre-assembled particle structure during prolonged illumination with circularly polarized light. The structure passes from tightly assembled through chain-like, ring-like, flower-shaped and dog-shaped stages before final disassembly. Scale bar (white): 5 µm. Green font indicates respective times from circularly polarized illumination onset. Note that, although some of these structural motives were repeatedly observed during the experiments, no deterministic control was obtained over such transformation sequences, in part certainly due to the varying particle sizes and initial assembly conditions. Full movies are shown in Supplementary Movie 7.

Supplementary Figure 10 – Particle size influence on capillary assembly-disassembly

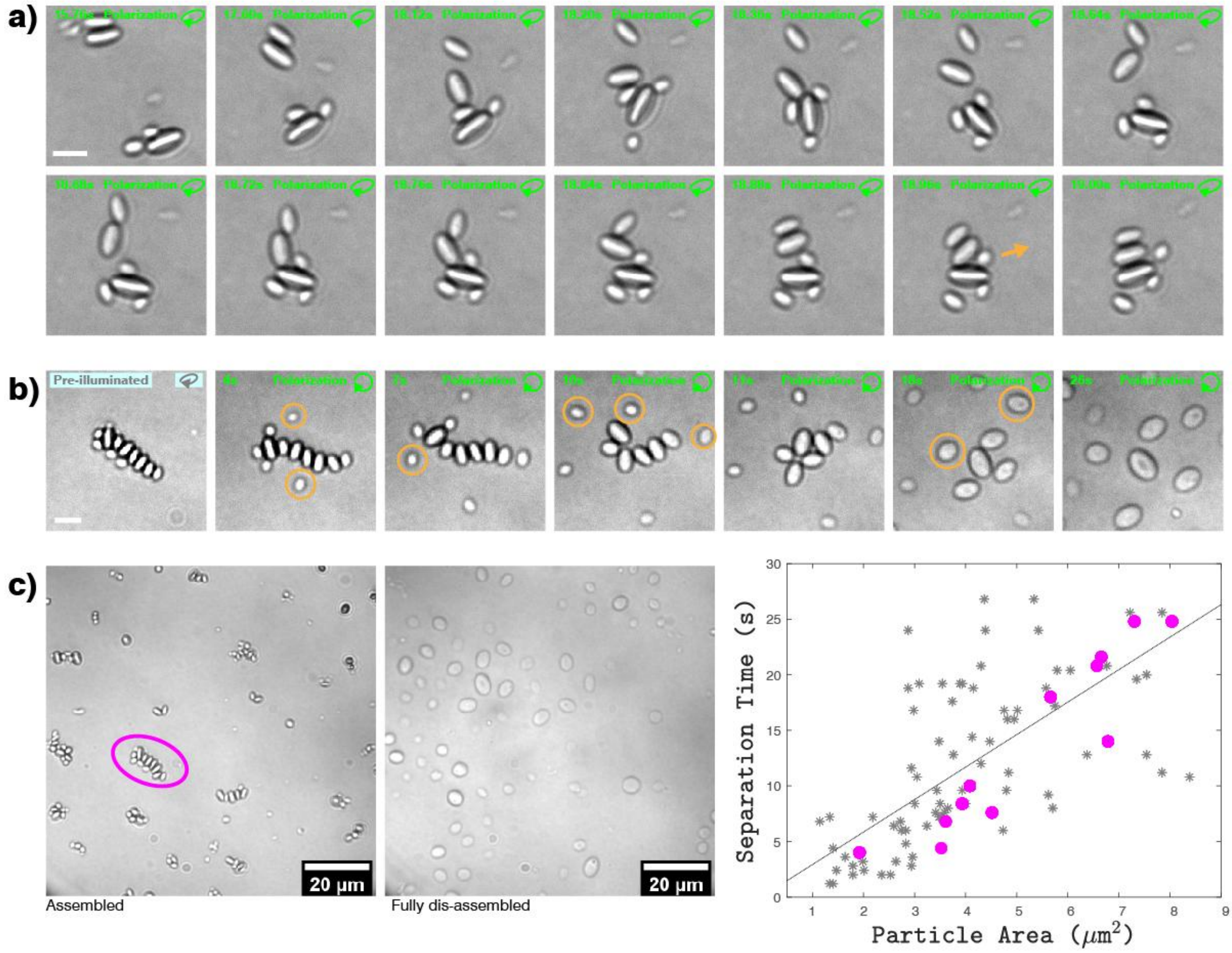

Supplementary Figure 10: a) Example of fast dynamics in a multi-particle assembly process under illumination with elliptically polarized light. The two largest particles in the field of view cause the largest interface deformations, giving rise to the strongest capillary interaction. Initially, the upper large particle leaves its partner to approach the lower large particle (top row). Upon contact it rebounds, before approaching once again, this time with its initial partner attached (bottom row). After rotating into parallel orientations, the two large particles squeeze out a small particle between them and assemble side-by-side, with the small particles located in the assembly periphery. The full sequence is shown in Supplementary Movie 8 (first part). Scale bar (white): 5 µm. Intensity: 219 W/cm^2. b) Example of the stepwise disassembly using circularly polarized light of a large multi-particle aggregate, which was previously assembled by elliptically polarized light. Due to assembly processes as shown in (a), the smallest particles are typically already located on the aggregate's periphery (left). During the illumination, these small particles dissociate from the aggregate rapidly, whereas larger particles first rotate into a tip-to-tip configuration, before also disassembling (similar to what can be seen on Supplementary Figure 9). Orange circles highlight particles that just left the aggregate in each frame. Scale bar (white): 5 µm. Intensity: 219 W/cm^2. c) Overview images showing the fully assembled and fully disassembled states, before and after illumination with circular polarization for 30 s of several assemblies, including the one in b (magenta outline) and the full sequence is shown in Supplementary Movie 8 (second part). On the right, the particle area is evaluated for all particles at the same timepoint (4 s after illumination onset) and plotted against the manually evaluated disassembly time, with the data shown in b colored in magenta. The data shows the values for 89 particles in total stemming from 13 different assemblies and displays a clear trend of increasing particle disassembly time vs. particle area (linear slope 2.9 s/µm^2, R2 = 0.4). Spread in the data obtained can be attributed to the polydisperse particle interactions, where not only a particle's size, but also the size of the particle it is dissociating from, play a role. The frame used for extracting the particles' areas, the employed imageJ procedure, the segmented frame and the extracted values are included in the shared data folder.

Supplementary Figure 11 – Extent of assembly after continuous flow

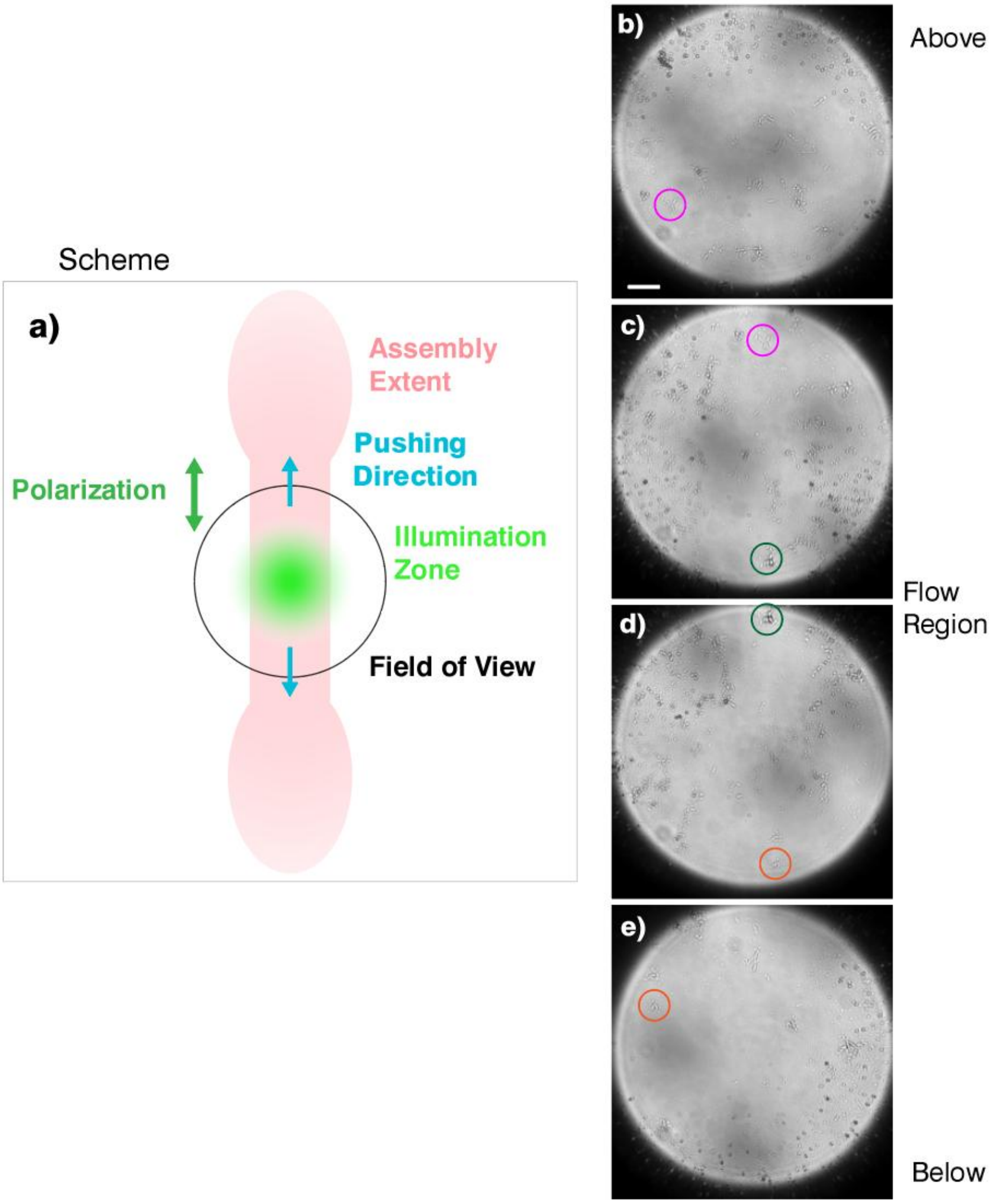

Supplementary Figure 11: a) Scheme of how the capillary assembly of particles extends beyond the field of view (here in y-direction for y-polarized illumination) after prolonged capillary assembly and deformation-driven flow. b-e) Roughly adjacent frames showing the areas above and below the illuminated zone in Figure 3 and Supplementary Movie 9, providing an overview over the assembled structure. Scale bar (white): 20 µm. Circles with same color label the same features to provide visual orientation. The illumination zone during the flow was smaller than the field of view and located in the lower region of (c) and the upper region of (d). The underlying movie of the experimenter moving the field of view across the final particle assembly, in which still no individual particle diffusion is observed and where stacking of larger particles can be perceived, is included in the shared data folder as:
'//Flow_Raw_Movies/001_M9_LargeParticles_YPOL_50fps_EXTRA_OV_afterFlow.avi'.

Supplementary Figure 12 – In situ rotation of the polarization/flow direction

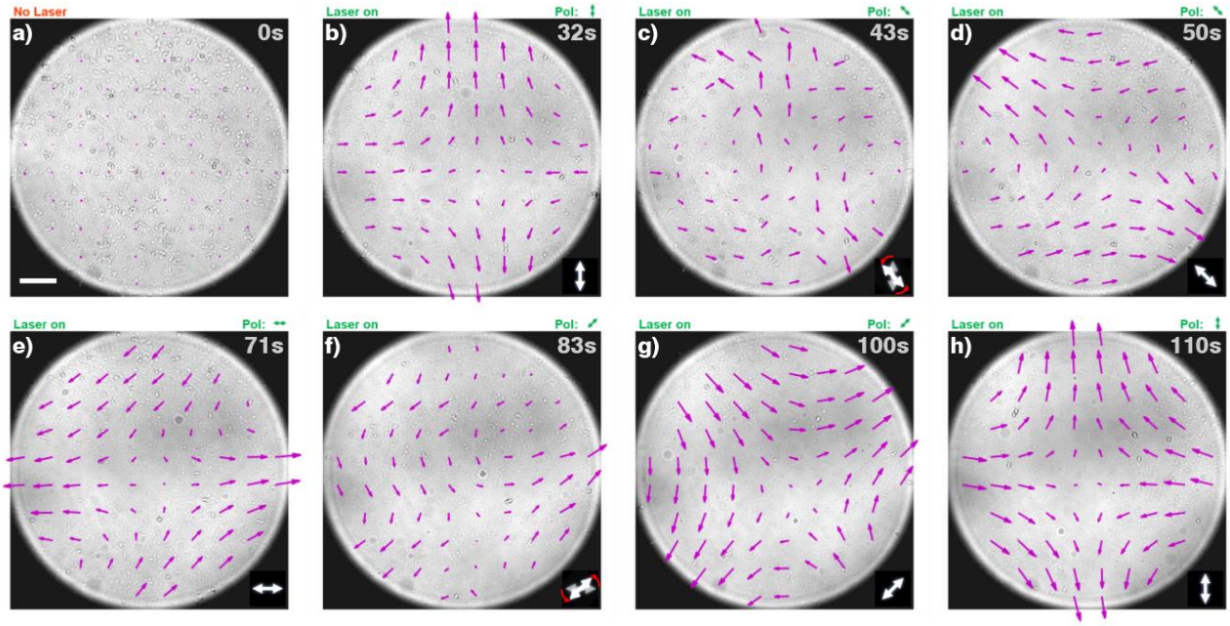

Supplementary Figure 12: a-h) Selected snapshots from Supplementary Movie 11 with overlaid flow-fields (magenta), showing how the flow direction follows the varying polarization direction during the same flow event. The time intervals of c and f include a discrete step of the illumination polarization by 45 degrees, giving rise to transient features. The time intervals used for flow-field computation and averaging are 3.3s. Scale bar (white): 20 µm.

Supplementary Figure 13 – Flow attempts with non-linearly polarized illumination

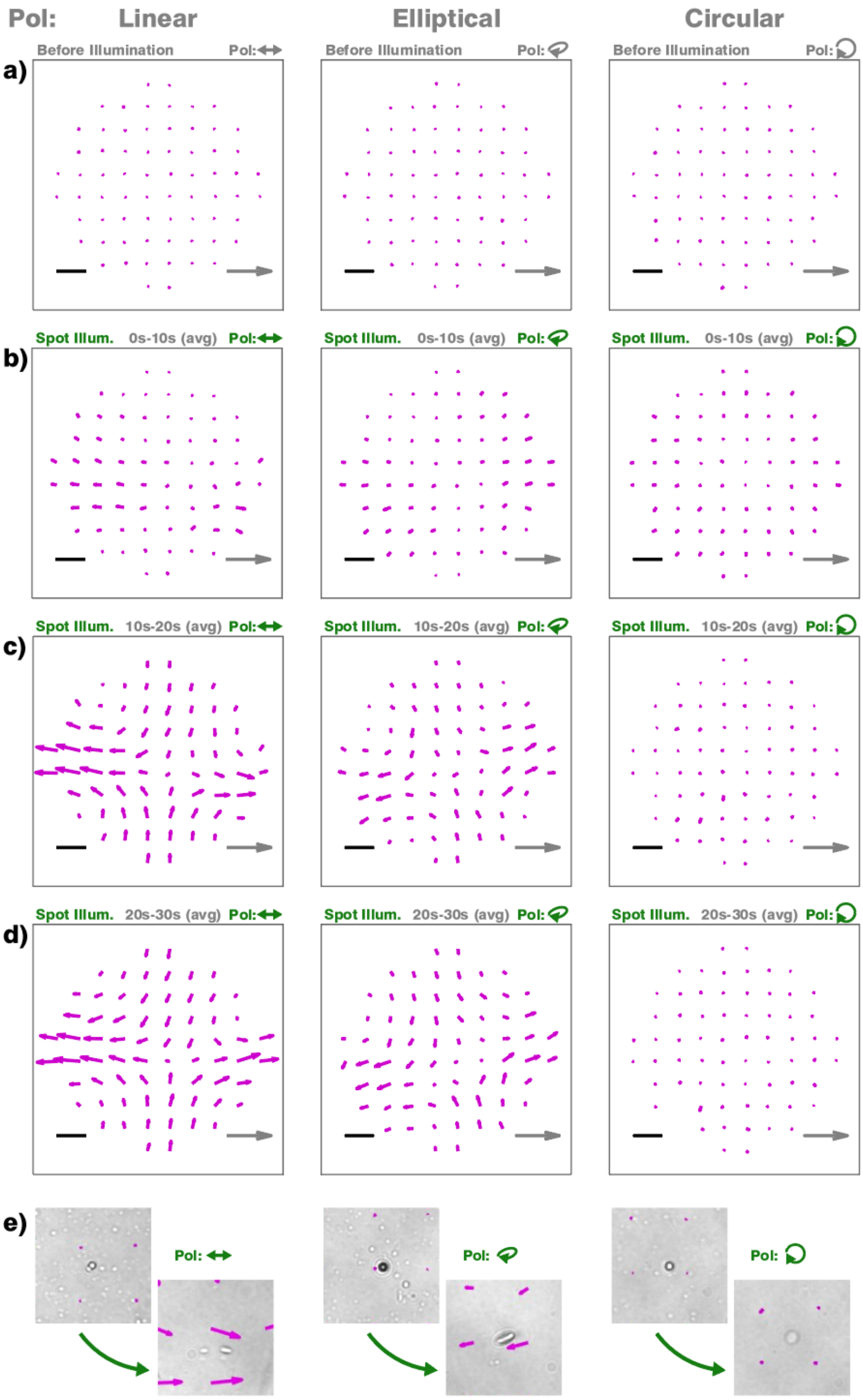

Supplementary Figure 13: Flow fields from the illumination events shown in Supplementary Movie 12, using linearly (left), elliptically (middle) and circularly polarized (right) illumination. Flow fields are shown before illumination (a) and averaged over adjacent 10 second intervals after illumination is turned on (b-d). Scale bars (black): 20 µm. Reference arrows (gray): 20 µm/s. While a tiny transient response is visible for all samples in b, no sustained flows develop for the circularly polarized illumination d (right), whilst linear polarization leads to the strongest sustained flow d (left). e) Zoomed snapshots from the movie at different timepoints showing a prominent particle before illumination and after illumination was turned on. The snapshots allow to recognize the characteristic deformation of the particles, particularly the disk-like shape under circularly polarized illumination. Intensity I = 56 W/cm^2.

Supplementary Figure 14 – Alternating circularly and linearly polarized illuminations.

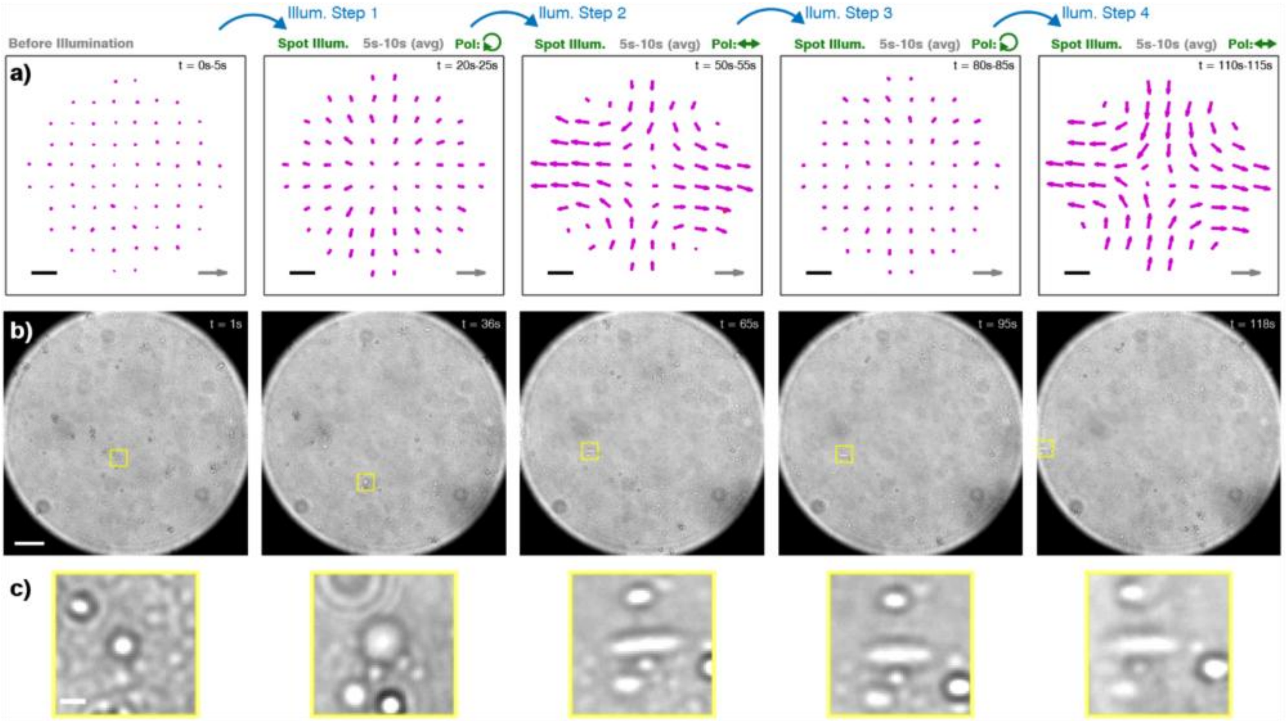

Supplementary Figure 14. a) Flow fields before (left) and during (towards right) sequential illumination steps with an alternatingly circularly and linearly *x*-oriented polarized laser. Only during illumination steps with linear polarization does a persistent outflow develop. Scale bars (black): 20 µm. Reference arrows (gray): 5 µm/s. Time *t* indicated in the upper right of the flowfields reports total movie time. b) Frames from the corresponding movie which is included in the shared data under '//Flow_Raw_Movies/015_Alt_CircHorizPol_50fps.avi'. The yellow rectangle tracks a prominent particle with easily appreciable deformations, which is shown in magnified insets in (c), during the sequential illumination steps. Scale bars (white): 20 µm in (b), 2 µm in (c). Notably, during illumination step 3, no persistent flow develops despite the particles being strongly pre-deformed along the *x*-axis after illumination step 2.

Supplementary Figure 15 – Flows with varying particle density

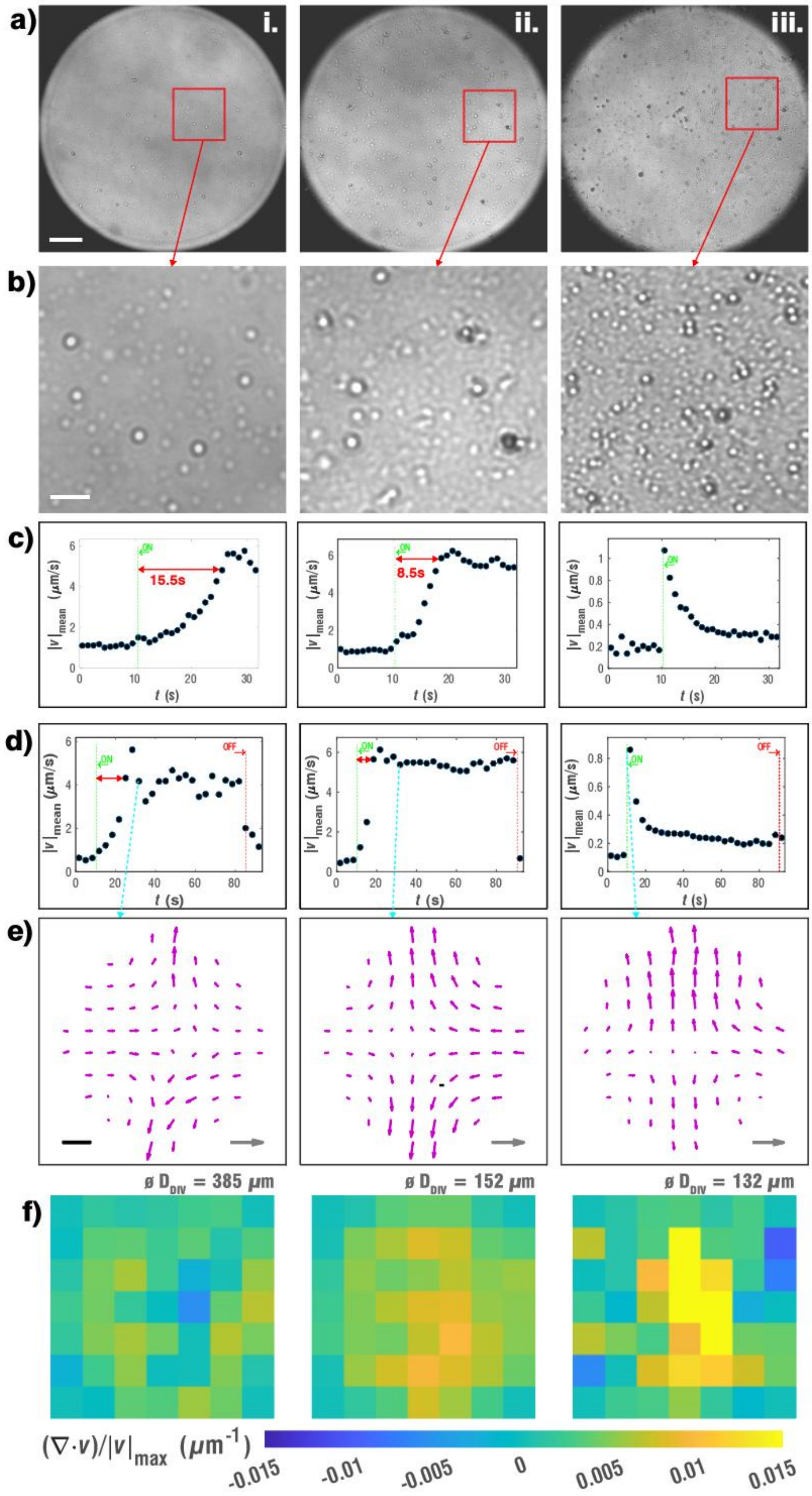

Supplementary Figure 15: a) Frames from flow movies (Supplementary Movie 13) with different particle densities increasing from left to right (i-iii, 5x concentration steps for the precursor solution). Note that in the highest density case

(iii), particle diffusion was severely hampered, as the particles formed a densely packed carpet. Scale bar (white): 20 µm. b) Insets for better visualization of the particle density differences. Scale bar (white): 5 µm. c) Temporal evolution of the mean of the individual absolute velocities in the flowfield during the first 20s of illumination (1s steps). A slightly slower rise is observed for the dilute sample (i), whilst the carpet-like sample (iii) deforms with maximal velocity immediately. d) Full length velocity graphs (3s steps), showing constant plateaus for the sustained flows, whilst the carpet's velocities rapidly decay (iii), owing to the saturating deformation. e) Selected flowfields, corresponding to the respective datapoint indicated by the cyan dotted arrow. Scale bar (black): 20 µm, reference arrows (gray): 20 µm/s (i), 20 µm/s (ii), 3 µm/s (iii). The outflow/inflow ratio seems to increase for higher concentrations (indicating more flow divergence). f) Relative divergence calculations over the field of view, averaged over 20 timesteps (3.3s) in each case, and displayed on the same color scale, confirming higher relative divergence for higher concentrations. The inverse of the average relative divergence (D_{Div}) is calculated omitting the outermost boarder values (non-illuminated) and indicated above each plot. This value estimates the characteristic distance over which divergence alone would build up to the maximal velocity present on the flowfield. Finally, note that both intensity and illumination spot size were slightly decreased for the most concentrated case (iii), to limit heavy absorption by the dense particles. Raw data from iii. was also rotated by 90 degrees to fit the flow direction of the other movies.

Supplementary Figure 16 – Stripe illumination with orthogonal/parallel polarization

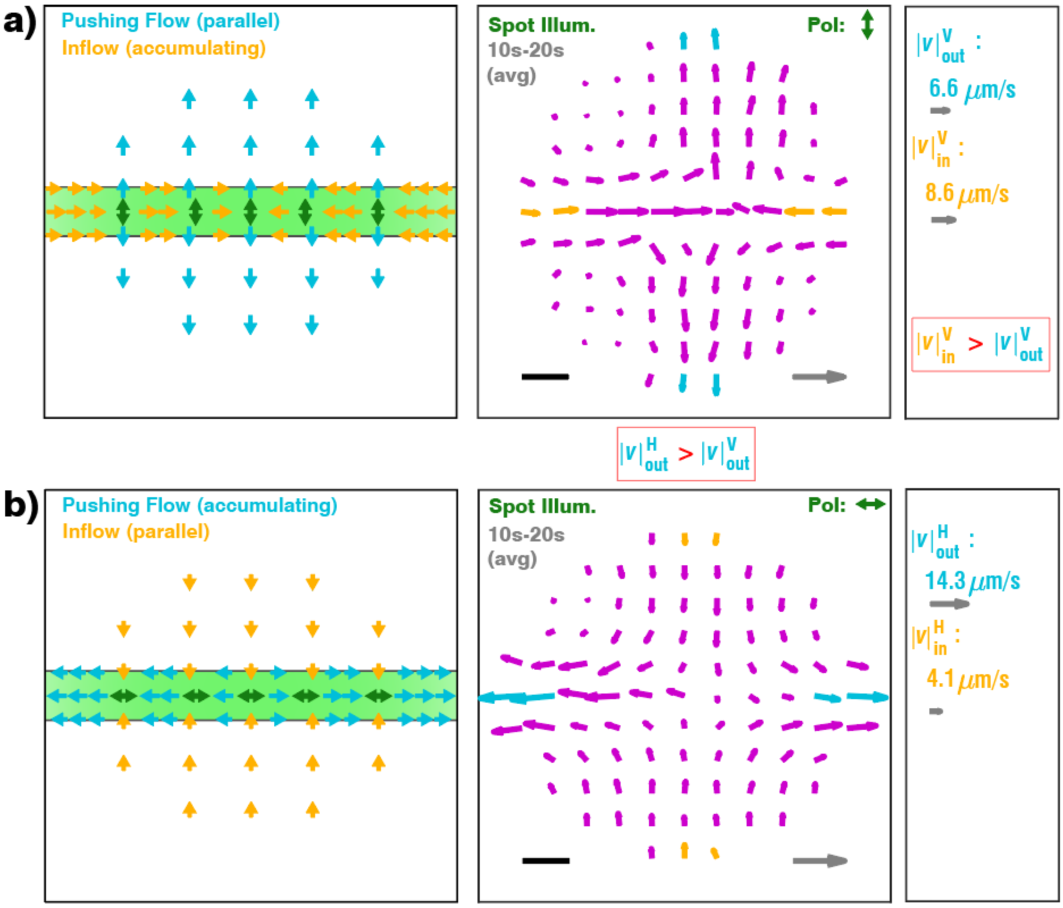

Supplementary Figure 16: a) Scheme (left) and flowfield (right) for a stripe illumination experiment with polarization oriented orthogonally to the stripe pattern, at low particle density (computed based on the data shown in Supplementary Movie 14). As schematically illustrated, the outflow perpendicular to the stripe illumination acts only over a short pushing distance (short axis of the stripe). The compensating inflow on the other hand accumulates along the stripe. In addition, the compensating inflow should be locally similar in size to the outflow everywhere, due to the low divergence condition. As a result, the average extremal inflow velocity (8.6 µm/s) surpasses the average outflow velocity (6.6 µm/s) in this configuration. b) Scheme (left) and flowfield (right) for the same illumination pattern, but with polarization oriented parallel to the long axis of the stripe. In this case, it is the outflow velocity that accumulates, which leads to a higher outflow velocity in this case (14.3 µm/s), consistent with a pushing effect acting over a much longer distance. All velocity averages are taken as the mean of the individual absolute velocities of the arrows marked in the corresponding colors on the respective plot. Scale bars (black): 20 µm. Reference arrows (gray): 20 µm/s.

Supplementary Figure 17 – Particles moving in opposite directions under shear flow

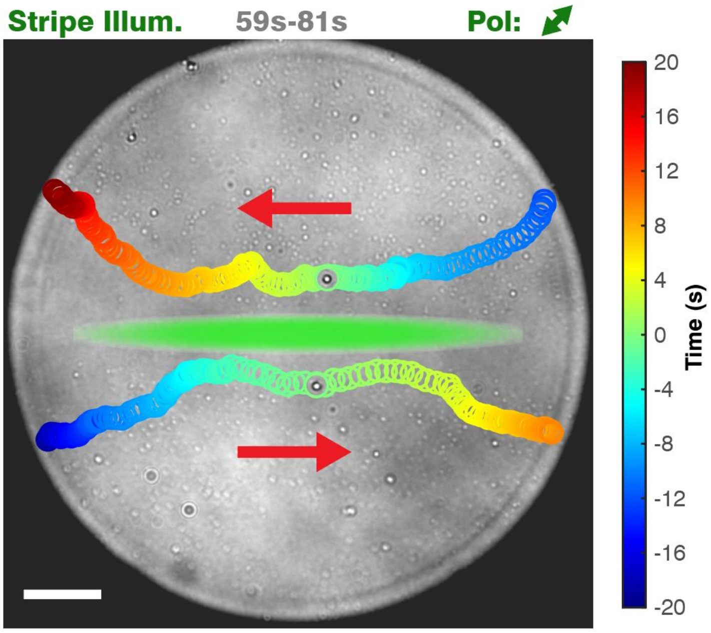

Supplementary Figure 17: Frame showing the field of view during the shear flow induced by diagonal polarization in Supplementary Movie 15 (right), at raw data time t = 70s, corresponding to 17.5 s in the accelerated movie. The trajectory of the two encircled prominent particles was tracked as they crossed the field of view in opposite directions (see Methods for the tracking procedure). The color bar to the right indicates the time difference between the particles' passage at a position labeled with a correspondingly colored circle, and the current frame time. Red arrows schematically depict the flow direction. Green overlay: schematic illustration of the stripe-shaped illumination. Scale bar (white): 20 µm.

Supplementary Figure 18 – Manual measurement of turbulent shear velocity

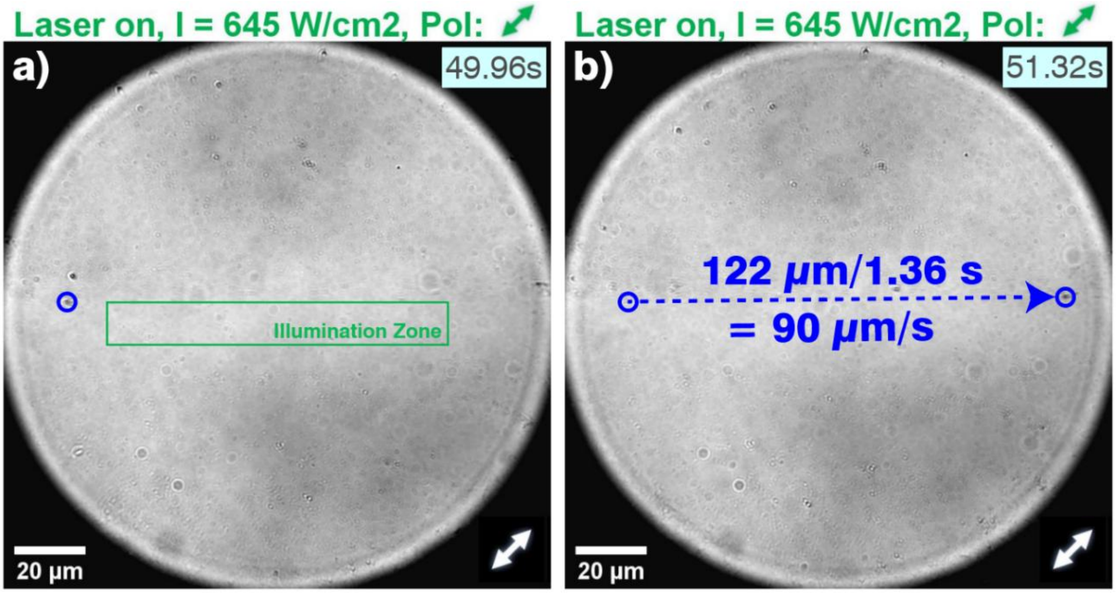

Supplementary Figure 18: Manual measurement to estimate the maximal translational velocities involved in the slightly turbulent shear flow of Supplementary Movie 16. The correlation-based algorithm, relying on spatiotemporal averaging (over a whole tile and a timestep), would perform poorer at this task. a) Movie snapshot showing a particle (inside blue circle) entering the field of view on the left. b) After traversing the field of view close to the upper border of the stripe-shaped illumination zone (green square), where the velocities are highest, the particle is again highlighted by a blue circle. From the distance travelled and the total time necessitated, the velocity for traversing the field of view can be estimated to 90 µm/s.

Supplementary Figure 19 – Evaluation of surface coverage

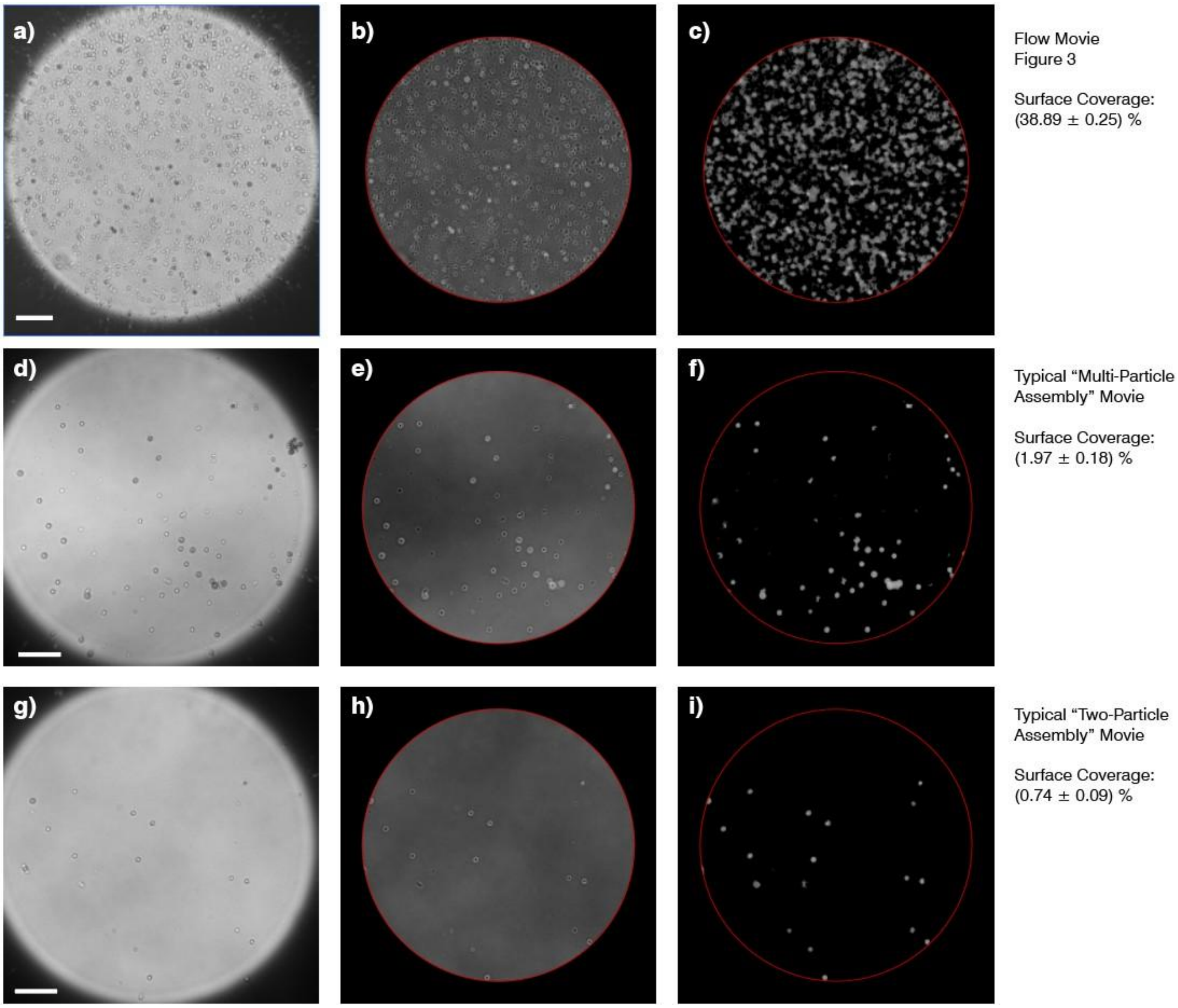

Supplementary Figure 19: The surface coverage was evaluated for movies in section 1-3 based on the following approach. First, 20 frames were selected from the initial seconds of the movies, where no illumination is provided yet (example images on the left). These frames were then inverted (example images, middle) after selecting the circular region of interest (red circle) and subsequently a particle localization with thresholding detection algorithm was applied. After locating the center of the particle, we obtained its area as the sum of all pixels surrounding the center that differ from the background intensity level (example images, right). The total fraction of the area occupied by detected particles was averaged over 20 frames, with mean values and standard deviation indicated to the right. a-c) Example images pertaining to Supplementary Movie 9, showing flow development described in section 3 (Figure 3). d-f) Example images pertaining to Supplementary Movie 7 (second part), showing an assembly-disassembly cycle involving multiple particles, described in section 2 (Supplementary Figure 9b). g-h) Exemplary images pertaining to Supplementary Movie 2, showing an assembly-disassembly cycle involving two particles, described in section 2 (Figure 2a). Coverage is significantly lower in all movies pertaining to the assembly-disassembly experiments than in movies used to assess the flow phenomenon. Scale bars (white): 20 µm.

Supplementary Figure 20 – Schematic algorithm steps in correlative velocity measurements

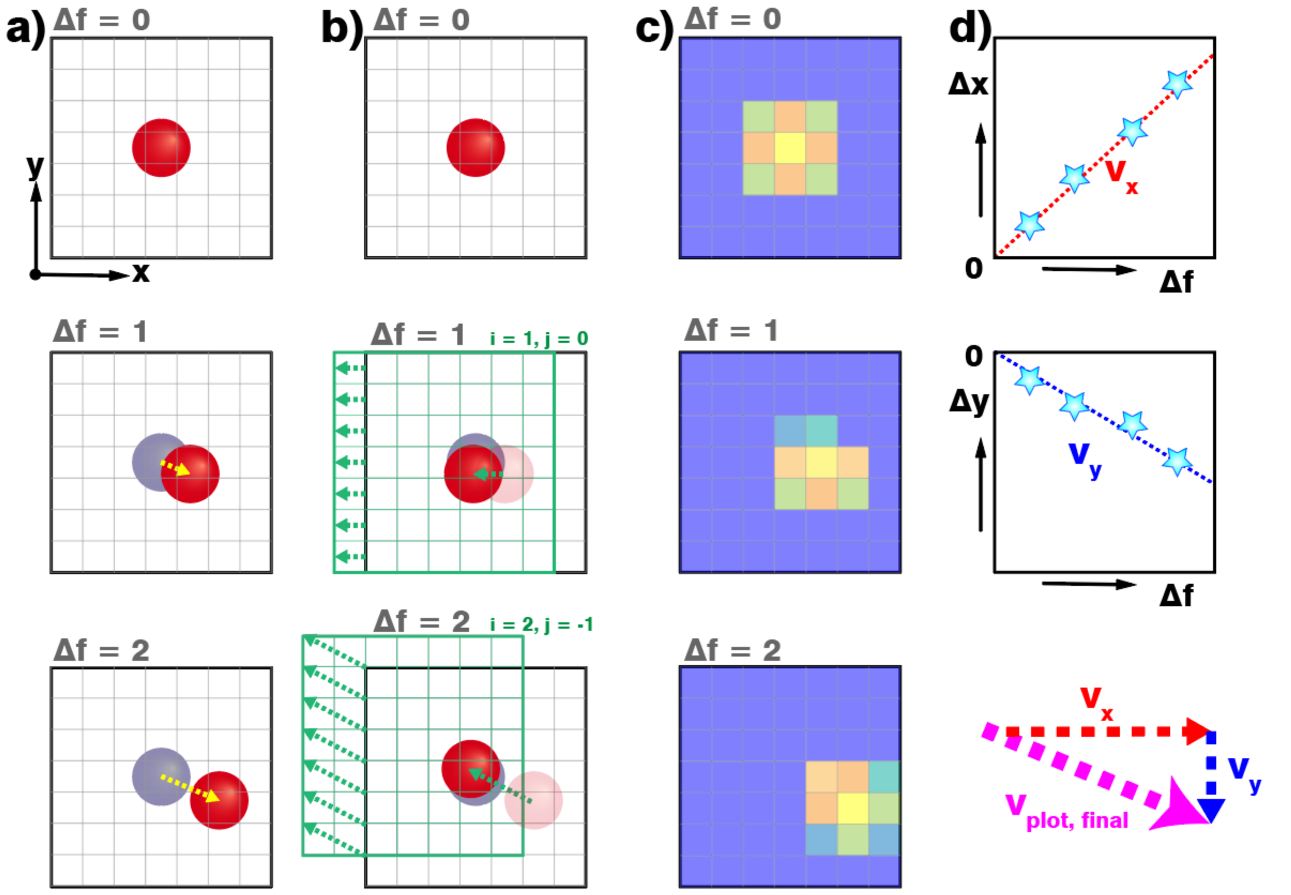

Supplementary Figure 20: Schematic representation of computation steps undertaken by the algorithm to extract the average drift velocity pertaining to a single tile and a single timestep. a) Sequence of consecutive movie frames showing the drift of a single particle (red) over three frames (top to bottom) and indicating its initial position ($\Delta f = 0$) as reference on the two later frames (gray). b) Spatially shifting the later images (bottom) by the right number of pixels, the drifting particle (red) can be almost exactly superposed with its initial position (gray). This will lead to the highest overlap on the correlative maps in c. c) Schematic correlative map examples where the maximum overlap shift displayed in b is naturally the brightest (yellow) pixel. However, the procedure is performed for all possible pixel shifts (up to a user-defined maximal shift value), in order to obtain full correlation maps. Further, the maps are taken as average, where any movie frame within the considered timestep is taken as reference frame exactly once, provided it is followed by at least Δf_{max} frames. The sequence of correlation maps for increasing Δf hence shows the averaged spatio-temporal drift-diffusion of features/particles. d) Schematic graph example plotting the *x*- and *y* positions of the correlation peak, which are obtained by Gaussian fitting of the maps shown in c. Finally, a linear fit of these positions will lead the average velocity along each axis (in pixel/frame units), permitting the construction of the final flow arrow after unit conversion.

Supplementary Figure 21 – Algorithm correlative velocity example on real data

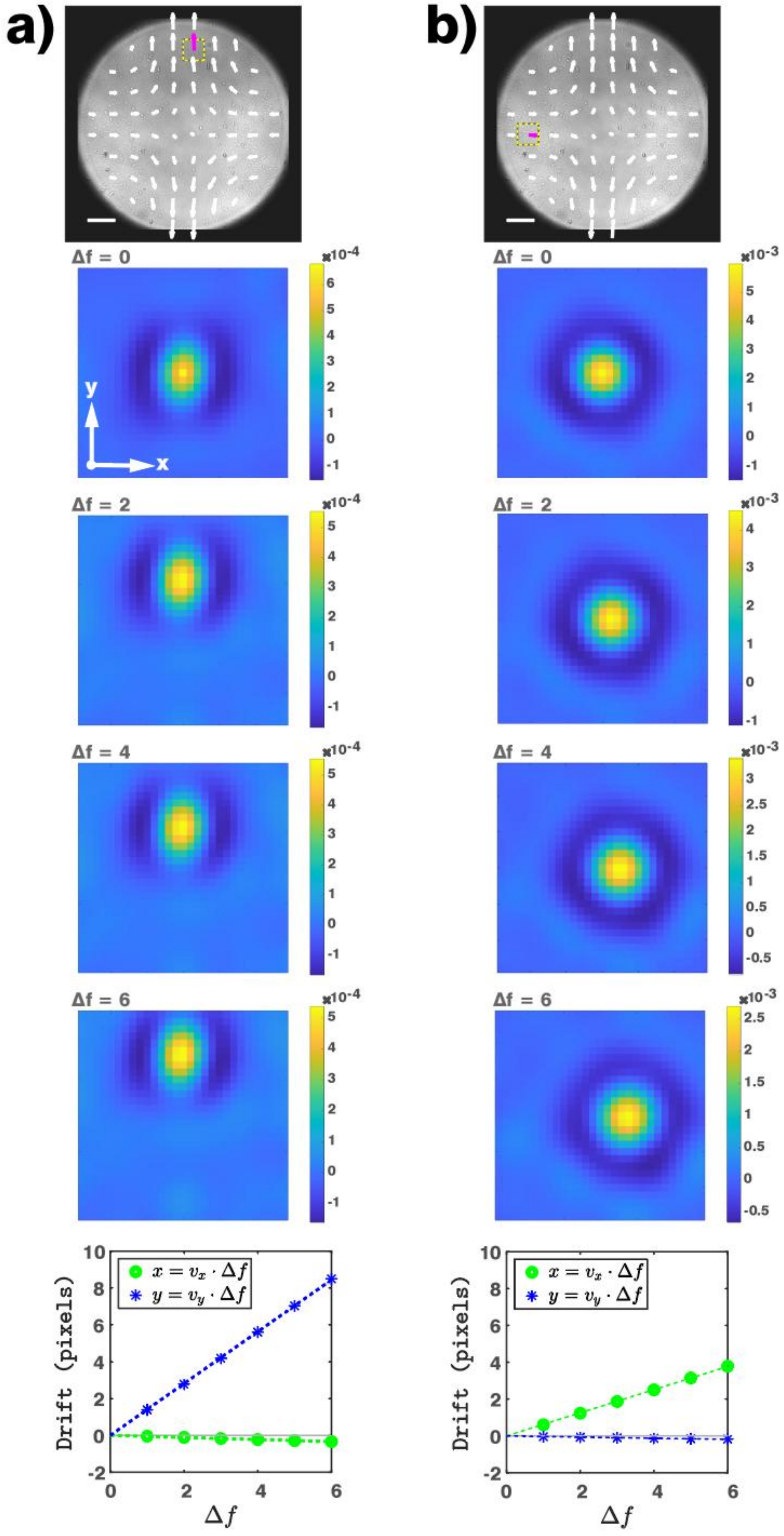

Supplementary Figure 21: Example of the computation of two flow vectors pertaining to the velocity field shown in Figure 4c (y-directed polarization flow). a) Computation of an arrow belonging to a tile where deformed particles are flowing out of the field of view at the upper border. b) Computation of an arrow in a tile where pristine, undeformed particles flow towards the zone of illumination. In each column, the images show (top to bottom): Flow field from Figure 4c with arrow to be computed marked in magenta and tile of interest marked by a yellow/black dotted frame (scale bar: 20 µm). The four computed correlation maps, corresponding to Δf = 0, 2, 4, and 6 respectively, which may be interpretated as the average motion of virtual particles initially located at the center (i.e., autocovariance at Δf = 0). Finally, a graph showing the (Gaussian fitted) x- and y-positions of the correlation peaks in Δf = 1-6, with linear fit of motion (dotted lines) used to extract the average drift velocity along each axis. Note that, whilst the correlation peak is perfectly isotropic in (b), and merely expanding due to the diffusion of pristine particles, the peaks in a can be seen to be slightly elongated in the y-direction. Although not used in this work, such information may be useful in other contexts. For example, the y-axis elongation of the spatial autocovariance function (Δf = 0) in (a) reflects the anisotropic shape of the y-axis deformed particles themselves. Another factor, apart from Fickian diffusion, which can influence the width and shape of the spatiotemporally evolving correlation peaks is velocity dispersion (i.e. non uniform velocity) either spatially within a tile, or temporally within a timestep (due to flow fluctuations).

Supplementary Figure 22 – Flowfield local divergence computations

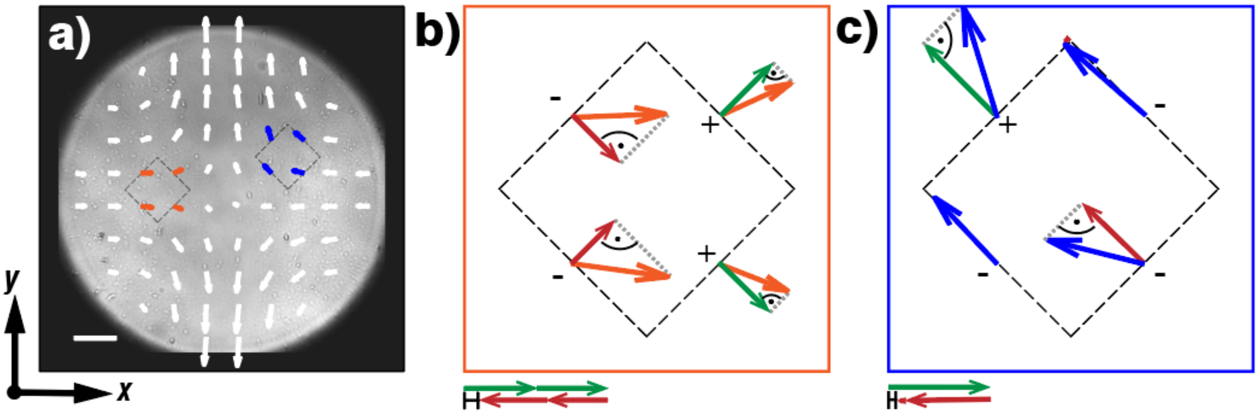

Supplementary Figure 22: Example of the computation of local divergence tiles such as shown in Supplementary Figure 15. a) Flowfield from Figure 4c (y-directed linear polarization flow). Two local divergence tiles are marked with black dotted squares (tilted at 45 degrees with respect to the flow vector grid), and the arrows corresponding to the tiles are colored (orange/blue). Scale bar (white): 20 µm. b) Computation of the local divergence averaged over the tile spanned by the orange vectors. Only contributions orthogonal to the tile boundary should be considered. Arrows representing negative contributions (net inflow) across the tile boundary are colored in red and marked with a minus sign, whilst positive contributions to the divergence (net outflow) are colored in green and labeled with a plus sign. At the bottom of the subplot, the length of the green and red arrows is compared. The difference between their total lengths is the measured (small) divergence, after correction by the factor (circumference/area). c) Same procedure applied to the tile spanned by the blue vectors.

SUPPLEMENTARY NOTE 1 – Coarse density variations and flow behavior

Protocol Adaptation

Using the method of simple pipette-aided dispersion of particles at the water-air interface (see methods), precise control over the density is difficult, and inter-sample (between different manual particle dispersions) as well as intra-sample (different areas of the same sample interface) variations are usually observed. Therefore, the density was varied in very coarse steps, by means of 1:4 sample pre-dilution, no dilution, and 5-fold sample pre-concentration prior to using the same interface deposition protocol. The three cases were compared side-by-side in Supplementary Movie 13 (Flow movies and live velocity graphs) and in Supplementary Figure 15 (Sample images, velocity graphs, flow field examples, divergence comparison). In case of the 5-fold, heavy, pre-concentration, a static film with no visible diffusion was observed on the interface. In both other case, diffusing particles are observed prior to illumination, with visually lower density for the strongly diluted sample.

Flow Rise and Persistence Comparison

In the two cases where the particles are not forming a static film, the upon illumination onset with linear y-axis polarization, a delay is observed, after which the polarization-driven flow emerges. We have attributed this delay to the necessity of first deforming particles and creating capillary particle assemblies, before the pushing mechanism can become effective at large scale (see section *Continuously deforming capillary assemblies*). In fact, as shown in Supplementary Figure 15c,d and apparent in Supplementary Movie 13, the non-diluted sample starts flowing after a shorter delay, possibly due to faster aggregation with more particles available. This picture is consistent with the case of the heavily pre-concentrated sample, where the particles form a static film. In fact, in this case the particles are already aggregated when the illumination starts and hence can start transmitting the symmetric deformation-caused pushing force immediately. Consequently, the deformation rate is maximal at the illumination onset and then follows the saturation-related decay that would be expected from the known deformation vs. illumination time behavior for azopolymer structures in fixed settings. Since the particles assume fixed positions with respect to each other in such a solid film, the movement ultimately stops, when the particles have been maximally deformed and no fresh particles can enter the illumination zone. This contrasts with the case of the two initially diffusing, lower density samples, where the sustained flow regime is obtained, and a constant flow velocity plateau is reached after the initial rise. We also note that in the dilute case, the flow is seen to be slightly more turbulent (and the plateau velocity less stable), possibly due to larger local particle density variations.

Divergence Comparison

Another parameter that can be computed from the flow fields and compared in these different situations is the two-dimensional flow divergence. Mathematically, the flow divergence is defined as the infinitesimal difference between local outflow and inflow in each spatial point. Although defined locally, the quantity may be integrated over an area, the result of which will quantify the total outflow/inflow difference across the boundary delimiting the integration area (divergence theorem). Hence, the average flow divergence may be estimated by simply comparing the total flow out of to the perpendicular flow into the illumination zone. A quick look at the exemplary flow fields in Supplementary Figure 15e shows that this difference seems to increase for higher particle densities (from left to right). This observation is confirmed by explicit computation of the divergence as shown in Supplementary Figure 15f. Here, the local average of the sustained, relative flow divergence in each zone between 4 adjacent flow-field arrows is mapped and displayed on identical color scales for all three situations (temporally averaging over the last 20 timesteps). The divergence is seen to roughly map the illuminating laser spot and increases for higher densities.

Assuming the water-air interface itself to be incompressible, one may hypothesize this difference in sustained divergence to stem from the average in-plane expansion - and hence from the deformation - of the particles themselves. In fact, also for a uniaxial, isochoric transformation associated with linear polarization, a net in-plane expansion results from the compression along the out-of-plane axis (optical axis), which is apparent from considering the deformation tensor describing such a transformation in the small strain approximation[34]:

$$\boldsymbol{F}^{\mathbf{uniaxial,isochoric}} = \begin{pmatrix} 1+\varepsilon_1 & 0 & 0 \\ 0 & 1+\varepsilon_2 & 0 \\ 0 & 0 & 1+\varepsilon_3 \end{pmatrix} = \begin{pmatrix} 1+\delta & 0 & 0 \\ 0 & 1-\frac{1}{2}\delta & 0 \\ 0 & 0 & 1-\frac{1}{2}\delta \end{pmatrix} \tag{1}$$

where the net in-plane area expansion is given by $\Delta A \approx \varepsilon_1 + \varepsilon_2 = \delta - \frac{1}{2}\delta = \frac{1}{2}\delta$. This considerable relative area change for deformation with linear polarization is also detected when quantitatively assessing the deformations in Supplementary Figure 4. Hence, if divergence is caused by this deformation-related in plane expansion of the particles themselves, it will naturally follow the local illumination intensity (i.e., the shape of the Gaussian illumination) and may increase for higher particle densities, as experimentally observed.

Finally, note that in the above, we have consistently compared the relative divergence $\nabla \cdot \mathrm{v}/|\mathrm{v}|_{\mathrm{max}}$, i.e. the divergence normalized by the maximum velocity present in the flow field. Given that the absolute divergence would linearly scale with the multiplication of all velocity vectors, this normalized

divergence is a more appropriate comparison tool between different flow situations with different speeds. In fact, its units (μm^{-1}) indicate that it describes a spatial frequency, which is the frequency with which a flow field with the given divergence and maximal velocity would spatially vary. Conversely, its inverse can be seen as the characteristic distance D_{Div} over which the measured divergence would (unidirectionally) cause velocity fields with the maximal amplitude in question. If this characteristic distance is much larger than the illumination zone (Gaussian spot size) in which the flow field is created, one may then conclude that divergence contributes in a negligible manner to the total flow field. The spatially averaged values for D_{Div}, computed over the illumination zone (25 innermost tiles), are indicated on Supplementary Figure 15f. One can see that in the lowest divergence case (left), this distance amounts to 385 μm, which is much larger than the illumination spot radius (<50 μm), and which thereby indicates that the divergence contribution to the flow field of such diluted samples can indeed be considered negligible. This opens for the obtention of a pure shear flow, which mathematically consists of curl contributions only, as shown in Figure 4 e-h.
