# Supplementary material for: Directional flows using capillary assembly of photo-deformable colloidal particles at water-air interfaces
Source: Nat Commun. 2025 Dec 24;17:1004. doi: 10.1038/s41467-025-67739-9 (PMC12847863; doi:10.1038/s41467-025-67739-9)
Supplement: Supplementary file 2 — Description of Additional Supplementary Files [file 41467_2025_67739_MOESM2_ESM.pdf]

Description of Additional Supplementary Files

Supplementary Movie 1

Movie showing the smooth deformation of azopolymer particles adsorbed to an air-water interface under illumination with linear (left), circular (middle), and elliptical (right) polarization at a wavelength of λ = 532 nm and an intensity of I = 219 W·cm^{-2}. Real time acquisition speed (non-accelerated).

Supplementary Movie 2

Movie showing the assembly of two azopolymer particles, initially freely diffusing while adsorbed to an air-water interface, upon deformation into ellipsoidal particles with elliptically polarized light (λ = 532 nm, I = 219 W·cm^{-2}). At a later stage, the particles are disassembled upon prolonged exposure to circularly polarized light, with otherwise identical illumination parameters. 5x accelerated.

Supplementary Movie 3

Movie showing the assembly of several azopolymer particles, initially freely diffusing while adsorbed to an air-water interface, upon deformation into ellipsoidal particles with elliptically polarized light (λ = 532 nm, I = 219 W·cm^{-2}). At a later stage, the particles are disassembled upon prolonged exposure to circularly polarized light, with otherwise identical illumination parameters. 5x accelerated.

Supplementary Movie 4

Movie showing the assembly of two azopolymer particles, initially freely diffusing while adsorbed to an air-water interface, upon deformation into rod-like particles with linearly polarized light (λ = 532 nm, I = 339 W·cm^{-2}). At a later stage, the particles are disassembled upon prolonged exposure to circularly polarized light, with otherwise identical illumination parameters. 5x accelerated.

Supplementary Movie 5

Movie showing the assembly of several azopolymer particles, initially freely diffusing while adsorbed to an air-water interface, upon deformation into rod-like particles with linearly polarized light (λ = 532 nm, I = 171 W·cm^{-2}). At a later stage, the particles are disassembled upon prolonged exposure to circularly polarized light, with otherwise identical illumination parameters. 5x accelerated.

Supplementary Movie 6

Movie showing the behavior of several azopolymer particles, freely diffusing while adsorbed to an air-water interface, upon deformation into disk-like particles with circularly polarized light (λ = 532 nm,

I = 219 W·cm^{-2}). Despite similar interparticle distances to Supplementary Movies 3, 5 and 7, no assembly is induced by the in-plane isotropic deformation and the particles continue to diffuse freely while they are being deformed. 5x accelerated.

Supplementary Movie 7

Two acquisitions added sequentially which show the assembly of multiple particles with linearly|elliptically polarized light, and subsequent disassembly using circularly polarized light (λ = 532 nm, *I* = 171|219 W·cm^{-2}). During disassembly multiple out-of-equilibrium configuration pathways can be seen. 5x accelerated.

Supplementary Movie 8

Two acquisitions added sequentially to show the influence of particle size on the assembly-disassembly dynamics. In the first part, the fast dynamics of multi-particle assembly under elliptically polarized illumination are shown (λ = 532 nm, *I* = 219 W·cm^{-2}). Larger particles push smaller particles aside to assemble. Real time acquisition speed (non-accelerated). The sequence is repeated in slow motion, 0.25x decelerated. In the second part, particles previously assembled using elliptically polarized illumination are disassembled using circularly polarized illumination (λ = 532 nm, *I* = 219 W·cm^{-2}). Smaller particles flatten sufficiently to disassemble from the aggregate before large particles do so. 2x accelerated.

Supplementary Movie 9

Movie showing larger particles (Supplementary Figure 1a) at high density under illumination with linear polarization along the *y*-axis (frame vertical). Upon exposure, the particles are seen to deform, assemble, and initiate a flow. When illumination is interrupted, the flow stops, and the particles appear rigidly assembled. Illumination parameters: λ = 532 nm, *I* = 100 W·cm^{-2}, Gaussian beam. 5x accelerated.

Supplementary Movie 10

Movie showing the sustained flow of smaller particles (Supplementary Figure 1b) under illumination with linear polarization along the *y*-axis (frame vertical) on the left and along the *x*-axis (frame horizontal) on the right. Computed flow-field arrows (magenta) are overlayed over the original microscope movie images. Illumination parameters: λ = 532 nm, *I* = 56 W·cm^{-2}, Gaussian beam. 4x accelerated.

Supplementary Movie 11

Movie showing the rotating flow direction of smaller particles (Supplementary Figure 1b) under illumination with linear polarization whose axis is rotated stepwise for a total of 180 degrees. Computed flow-field arrows (magenta) are shown besides the original microscope movie images. Illumination parameters: λ = 532 nm, I = 26 W·cm^{-2}, Gaussian beam 3x accelerated.

Supplementary Movie 12

Movie showing the behavior of smaller particles (Supplementary Figure 1b) under illumination with linearly, elliptically and circularly polarized illumination polarization (left to right). Computed flow-field arrows (magenta) are overlayed over the original microscope movie images. Sustained flows occur only for linear and elliptical polarizations and are strongest for the linear polarization case. Illumination parameters: λ = 532 nm, I = 56 W·cm^{-2}, Gaussian beam. 4x accelerated.

Supplementary Movie 13

Movie showing the flow of smaller particles (Supplementary Figure 1b) under illumination with linearly polarized light. Left to right: particles initially filling the interface with very low density, intermediate density, and fully filled packed layer. In the top graphs, the mean absolute velocity over all arrows for the different situations is tracked. Illumination parameters: λ = 532 nm, I = 56|56|32 W·cm^{-2}, Gaussian beam. 2x accelerated.

.

Supplementary Movie 14

Movie showing the flow of smaller particles (Supplementary Figure 1b) under a stripe-shaped illumination with linear polarization along the *x*-axis (parallel to stripe) on the left and along the *y*-axis (perpendicular to stripe) on the right. Illumination parameters: λ = 532 nm, I = 645 W·cm^{-2}, stripe-shaped pattern. 2x accelerated.

Supplementary Movie 15

Movie showing the flow of smaller particles (Supplementary Figure 1b) under a stripe-shaped illumination with linear polarization offset by +45 degrees (counterclockwise, with respect to stripe axis) on the left and by -45 degrees (clockwise, with respect to stripe axis) on the right. Illumination parameters: λ = 532 nm, I = 645 W·cm^{-2}, stripe-shaped pattern. 4x accelerated.

Supplementary Movie 16

Movie showing the flow of smaller particles (Supplementary Figure 1b) under a stripe-shaped illumination with linear polarization offset by +45 degrees (counterclockwise, with respect to stripe axis)

and density slightly increased with respect to Supplementary Movie 12, giving rise to faster flows and slight rotational turbulences. Illumination parameters: λ = 532 nm, I = 645 W·cm^{-2}, stripe-shaped pattern. Real time acquisition speed (non-accelerated).
